# Assessing anxiety symptom severity in Rwandese adolescents: cross-gender measurement invariance of GAD-7

**DOI:** 10.3389/fpsyt.2024.1346267

**Published:** 2024-03-11

**Authors:** Lisa Cynthia Niwenahisemo, Su Hong, Li Kuang

**Affiliations:** Department of Psychiatry, The First Affiliated Hospital of Chongqing Medical University, Chongqing, China

**Keywords:** multiple group comparison, measurement invariance, psychometric properties, generalized anxiety disorder (GAD-7), Rwanda, adolescents

## Abstract

**Background:**

Anxiety disorders are among the most common mental health problems experienced by adolescents worldwide because of their evident significant impact on their quality of life and functioning. The generalized anxiety disorder item (GAD-7) was manufactured to identify the severity of self-reported anxiety symptoms. Efforts to address and screen for mental health problems in Rwanda have been limited, and the importance of screening for anxiety disorders is high. The primary aim of this study was to analyze the psychometric properties of the Kinyarwanda version of the Generalized Anxiety Disorder GAD-7, and then test the measurement invariance of the GAD-7 by gender.

**Methods:**

We used the Rwandese version of GAD-7 among secondary school students in Kigali city (n=1813). Measurement invariance of the GAD-7 across gender and report on anxiety symptom severity prevalence. Exploratory factor analysis (EFA) and confirmatory factor analysis (CFA) were used to examine measurement invariance.

**Results:**

Our findings demonstrated that in the sample of 1813 adolescents aged between 12 and 17 years, generalized anxiety symptoms prevalence rates were higher in females (46.4%) than males (n= 29.8%) GAD-7 demonstrated good reliability and validity coefficients with a Cronbach’s α of .077 and KMO and Bartlett test of Sphericity = 0.835. In addition to these psychometric properties, the GAD-7 screening scale had equivalence for configural and metric invariance across groups with excellent fit indices, and we confirmed partial scalar invariance across groups.

**Conclusion:**

The GAD-7 can be used in cross-group comparison of generalized anxiety disorder prevalence, and we acknowledge that full scalar invariance is generally difficult to confirm, especially due to gender differences. We recommend that future studies further investigate populations living in rural areas and conduct trials that will focus on anxiety-specific treatment in Rwandan Clinical health care centers to determine the diagnostic accuracy of this screening tool.

## Introduction

Global research studies estimate that at least 10%–20% of children and adolescents throughout the world have mental health problems (1), and anxiety disorders are among the most common mental health problems experienced by adolescents (2, 3). The global burden of disease GBD estimated that anxiety disorders contributed to 26.8 million disability-adjusted life years in 2010 (4), and more studies found that negative life events were associated with an increased risk of anxiety disorders in adolescents (3). Anxiety disorders are characterized by excessive and persistent fear or worry that interferes with daily activities. They can manifest in several ways, including generalized anxiety disorder (GAD), panic disorder, social anxiety disorder (SAD), obsessive-compulsive disorder (OCD), and posttraumatic stress disorder (PTSD) (5).

Studies in sub-Saharan African countries have shown high rates of mental disorders such as posttraumatic stress disorder (PTSD), anxiety, and depression, ranging from 20% to 60% (6). Rwanda is one of the sub-Saharan countries with a population of approximately 13.5 million with at least 63% of the population being under the age of 25 (7). The country has come a long way since the 1994 genocide against Tutsi, which killed over one million people, leaving the country devastated and subjected to an inordinate level of both physical and psychological disability, giving rise to unprecedented numbers of mental disorders among both children and adults (7–10). The traumatic events of the Genocide led to a considerably high prevalence of mental health problems, and healthcare systems were destroyed (7). Research shows that children may also be affected by a parent’s suffering from mental disturbances (11). Rwanda Biomedical Center further illustrates gender differences, suggesting that 13.5% of the population suffers from anxiety disorders, with women being more affected than men (12).

Efforts to address mental health problems in Rwanda have been limited. In an attempt to support initiatives that would help in rapid screening, diagnosis, and treatment of mental illnesses, Rwanda embraced the principles of the Mental Health Gap Action Programme (mhGAP). However, regardless of this progress, there have been hindrances in meeting its ideals of adequate and timely provision of mental healthcare to all in need. There have been reports that the national budget for mental healthcare and the ratio of public mental health providers to the population within each district remain very low (13). Research has shown that most mental health problems begin in childhood and adolescence and suggests that budget saving is in mental health interventions including screening and treatment if delivered early through primary prevention and primary care (14). The need to screen for anxiety is accompanied by the knowledge that early childhood anxiety often interferes with social, emotional, and academic development (15), which most likely inspires involvement in substance abuse, suicide, educational achievement, and functional impairment (16). Anxiety disorders in children and adolescents cannot easily be assessed with standard questionnaires or interviews that have been derived from adult instruments (17), which highlights the need to validate available instruments in children and adolescents to ensure their diagnostic ability. This is rather crucial because it can be difficult to identify generalized anxiety disorder in adolescents, and they may be hesitant to talk about their worries and may not recognize that their symptoms are related to a mental health disorder bearing in mind that parents and teachers may also overlook the symptoms of generalized anxiety disorder, mistaking them for normal teenage behavior. The Generalized Anxiety Disorder Item (GAD-7) was manufactured to identify the severity of self-reported anxiety symptoms (18). The GAD-7 items follow the original DSM-IV diagnostic criteria for the aforementioned disorder (19). The accuracy of GAD-7 in detecting generalized anxiety disorder is adequate, and reliable screening leads to effective treatment. The adult version of GAD-7 has been used to screen for anxiety in adolescents in different countries, and evidence shows that this instrument is eligible to detect GAD symptoms and differentiate between mild, moderate, and severe GAD symptoms in adolescents and could be more efficient than other instruments (20–22).

While Rwanda already has an adult Kinyarwanda version of the GAD-7 as a sound instrument to evaluate anxiety symptoms, so far we do not have a validated GAD-7 instrument among adolescents in Rwanda. Therefore, we are conducting this research to assess whether this screening tool has good psychometric properties in adolescents and investigate if the one-dimensional model is appropriate across both genders. This cross-gender investigation was based on this comparability in age, psychosocial aspects, and educational status. The investigation of cross-gender equivalence of GAD-7 will have high relevance to the diagnosis of anxiety disorders and will serve as a prerequisite for other comparisons. This cross-sectional survey aimed to examine the psychometric properties of the Rwandese version of GAD-7 among secondary school students and assess the gender differences concerning the construct’s ability to determine anxiety symptoms severity. Before the study, we set two hypotheses: (1) the factor structure of GAD-7 will be equivalent across both genders in Rwandese adolescents, and (2) anxiety prevalence rates will not differ significantly between male and female Rwandese adolescents.

## Methods and design

### Sampling and data collection

We recruited the population of secondary students in Rwanda, through a convenience sampling method. The sample size was calculated using G*Power software (latest Ver. 3.1.9.7) (23, 24). We included participants according to whether they were aged between 12 and 17 years, were currently enrolled in school at the moment, had no previously diagnosed mental health conditions, and had Kinyarwanda language proficiency to understand questions on the scale. Participants were excluded if they had a preexisting mental disorder, a learning disability that may impact their ability to participate, or cognitive impairments that could hinder their comprehension of the study materials.

The data were collected in Rwanda from November 2022 to January 2023, and participants from five secondary schools in Kigali used the Kinyarwanda version of the Generalized Anxiety Disorder Symptom Severity Scale (GAD-7) by manually distributing survey copies on site. The questionnaires took an average of 15 min to be completed by participants whose guardians have signed the consent form allowing them to take part in the survey.

### Generalized anxiety disorder

The GAD-7 questionnaire was designed by Spitzer et al. and published in 2006; it is quick to administer and is now used in research and clinical settings. The items in the scale enquire about the severity of the patient’s symptoms including target questions on nervousness, anxiety, being on edge, unable to stop worrying excessively, trouble relaxing, restlessness, irritability, and a constant fear that something horrible might happen with a specification to the past 2 weeks. Each item ranges from 0 to 3 on a 4-point Likert scale (0 = not at all, 1 = several days, 2 = more than half the days, and 3 = nearly every day). The addition of the scores of all seven items yields the GAD-7 total score, which ranges from 0 to 21. According to previous research studies, cutoff points ≥5, ≥10, and ≥15 based on the receiver operating features analysis for GAD-7 represent mild, moderate, and severe anxiety symptoms, respectively (19).

This questionnaire has been proven valid and reliable for use as a screening material and detecting the severity of symptoms in general populations as well as in primary care (25, 26); it was translated to Kinyarwanda using a structure approach of back-translation technique combined with bilingual technique (27) by the Integration of HIV Care into Mental Healthcare Services Technical Working Group from English to Kinyarwanda in 2011.

### Statistical analysis

The data were analyzed using IBM Statistical Package for the Social Sciences (SPSS) software version 26 (28). The exploratory factor analysis (EFA) was conducted to determine the construct validity and factor loadings of the items and determine factor structure of GAD-7. The internal consistency of the construct was measured using Cronbach’s alpha, and the construct validity was analyzed with the applicability of the Bartlett Test of Sphericity and Kaiser–Meyer–Olkin (KMO).

We used SPSS AMOS version 26 to perform a confirmatory factor analysis (CFA) model of the GAD-7 through which the indicators estimated and established the fit of a baseline model for GAD-7 in our sample. Following that, we conducted an analysis and interpretation of the models to achieve the study objectives by measuring Configural to confirm the consistency of the GAD-7 factor structures between groups where no equality constraints were imposed on the Configural model (29). We conducted the metric invariance as a prerequisite for making valid group comparisons to see whether factor loadings for each GAD-7 item were equivalent across groups (30), and last but not least, scalar invariance was done to verify whether mean item-level differences were fully explained by mean facto-level differences by testing for equivalent item intercepts (31).

In justifying our baseline model, we considered fit indices (root mean square error of approximation [RMSEA], chi-square minimum (CMIN (DF), Tucker–Lewis index [TLI], and comparative fit index [CFI]), standardized root mean square residual (SRMR), the normed fit index (NFI), and current anxiety theories. To assess model fit, we used the following guidelines: For absolute fit indices (RMSEA) and their 90% confidence interval, exact fit ≤0.001, close fit = 0.01–0.05, acceptable fit = 0.05–0.08, mediocre fit = 0.08–0.10, and poor fit = greater than 0.10; for relative fit indices (TLI and CFI), exact fit = 1.00, close fit = 0.95–0.99, acceptable fit = 0.90–0.95, mediocre fit = 0.85–0.90, and poor fit = less than 0.85 (Hu & Bentler, 1999); and last but not least, SRMR values <0.05 indicate a close fit whereas that between 0.05 and 0.10 is considered acceptable fit (32).

## Results

### Descriptive analysis

After data collection among the sample size of 1,813 Rwandan adolescents recruited from middle and high schools in Kigali city aged between 12 and19 years, with the following characteristics, female 885 (48.8%) and male participants account for 928 (51.2%) with age mean M = 15.80 and standard deviation SD = 1.90. According to the recommendations made by Kline (33) regarding the cutoff values (skewness ≤3, kurtosis ≤8), demonstrated a normal distribution (.786,.093) was demonstrated in the sample. In addition, we computed the prevalence of symptom severity, and the results showed that generalized anxiety disorder symptoms were more prevalent in women than in men; the severity of symptoms was 861 (47.5%), 582 (32.1%), 277 (15.3%), and 92 (5.1%). Following a cutoff point of >8 for the GAD-7 scale, anxiety symptom severity was higher in women at 46.4% than in men at 29.8%. One-way ANOVA tests were conducted and exhibited significant variation in measures for gender (F = 73.04, P <.001), and an independent t-test showed that women differed significantly from men (5335.25, P <.001) in generalized anxiety severity prevalence P <.001 (**Table 1**).

**Table 1:** Participant characteristics and anxiety prevalence.

	N (%)	M	95% CI	SD	F (sig.)	χ2	Skew	Kurt	Min	Mild	Mod.	Sev.
**Total**	1,813 [37.9%]	5.79	[5.57,6.00]	4.57			.786	.093				
**Female**	885 [53.6%]	6.71	[6.40,7.02]	4.70	73.04 P<.001				339	306	181	96
**Male**	928 [29.8%]	4.91	[4.63,5.18]	4.28					522	276	58	34
**Age**	_	15.80		1.90	_		-.294	-.637				
**GAD-7**	1813	.78			5,335.25 P<.001	71.76 P<.0001	.885	-.138	861 [47.5%]	582 [32.1%]	277 [15.3%]	92 [5.1%]

GAD-7, Generalized Anxiety Disorder; n, number of participants; M, mean; 95% CI, 95% confidence interval; SD, standard deviation; skew., skewness; kurt., kurtosis; min, minimal symptoms; mild, mild symptoms; mod., moderate symptoms; sev., severe symptoms; χ2, Pearson’s chi-square, P <.001.

Higher scores indicate greater self-reported symptoms of anxiety.

### Psychometric properties

**Figure 1:**
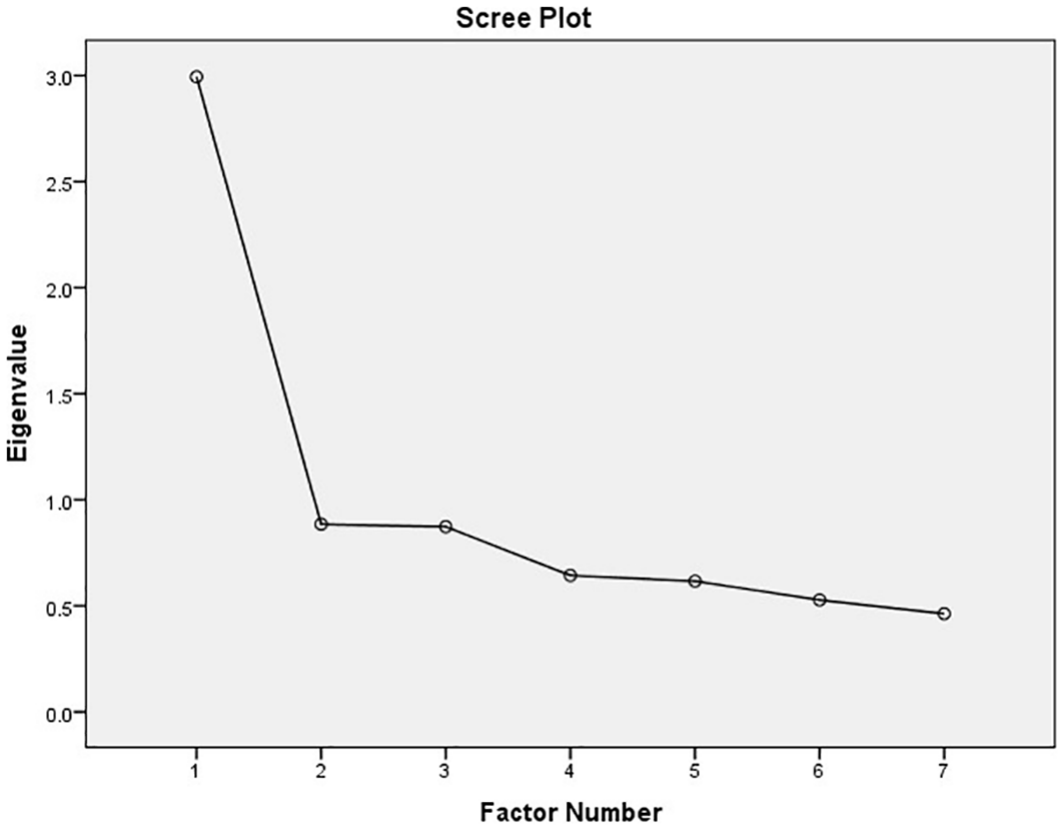
Scree plot.

The construct validity of GAD-7 was tested using KMO and Bartlett Test of Sphericity to check construct validity. The KMO coefficient was 0.835, which was considered excellent, whereas the Bartlett Test of Sphericity was found statistically significant (χ^2^ = 2,711.352, df = 21, P < 0.001) (**Table 2**), indicating that a factor analysis can be conducted for GAD-7. The item characteristics are summarized in (**Table 3**), mean (SD) values of the GAD-7 scale were 5.79 (4.579), and the mean values of the items ranged from 0.49 to 1.10. The internal consistency of GAD7 is α = 0.77. The Cronbach’s alpha if items were deleted were not higher than the overall Cronbach’s, and all intercorrelations between GAD-7 items were significant at P < 0.01, so all items were worth retention. Exploratory factor analysis was used to investigate the factor structure of the Kinyarwanda version of GAD-7, which yielded one factor being extracted as indicated by the eigenvalue and the scree plot inspection, which accounted for 42.80% of the total variance of GAD-7 (**Table 3** and **Figure 1**) all the items of the Kinyarwanda version of GAD-7 had statistically significant loadings whose values were greater than 0.5; hence, all items in the construct are important to interpret. Based on the exploratory analysis results, a one-factor model was analyzed for confirmatory factor analysis.

**Table 2:** KMO and Bartlett’s test of Sphericity.

Kaiser–Meyer–Olkin Measure of Sampling Adequacy		.835
Bartlett’s Test of Sphericity	Approx. ChiSquare	2,711.35
	Df	21
	Sig.	.000

DF, degree of freedom; sig., significant.

**Table 3:** Item characteristics and reliability.

GAD-7 Items	M	SD	Item total correlation	Corrected Item total-correlation	Factor loadings	Cronbach’s α
1. Feeling nervous or on edge?	.89	1.008	.680	.531	.694	.736
2. Not being able to stop or control worrying?	.74	1.018	.729	.595	.752	.723
3. Worrying too much about different things?	1.10	1.090	.728	.582	.735	.725
4. Trouble relaxing?	.58	.893	.609	.463	.626	.750
5. Being so restless that it is hard to sit still?	.49	.863	.581	.435	.587	.755
6. Becoming easily annoyed or irritable?	.90	1.076	.571	.379	.519	.769
7. Feeling afraid as if something awful might happen?	1.08	1.072	.653	.484	.633	.746
**GAD-7 total score**	5.79	4.579	1	_	_	.772

GAD-7, Generalized Anxiety Disorder 7-item; M, mean; SD, standard deviation; Cronbach’s αis the reliability/internal consistency.

### Confirmatory factor analysis

#### Measurement invariance of GAD-7

##### Single-group CFA

**Figure 2:**
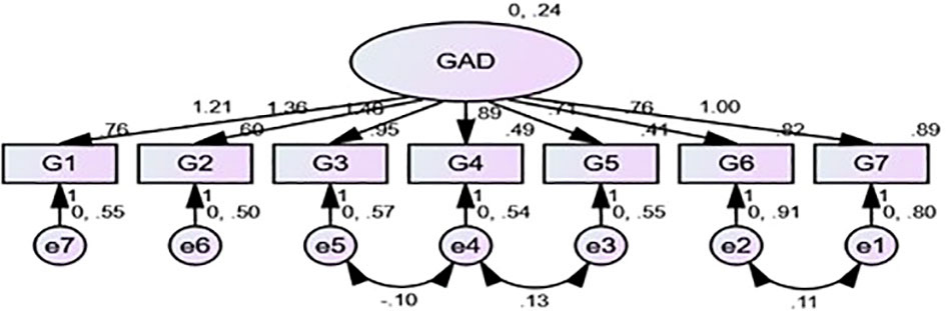
Factor structure for GAD-7.

The original one-factor model (**Figure 2**) was proven to fit according to the analysis from exploratory factor analysis and is demonstrated in the scree plot (**Figure 1**). The confirmatory factor analyses of CFI, RMSEA, and SRMR values were not very good (CFI = .940, RMSEA = .08 95% CI (.041,.119), and SRMR = .048); additionally, the CMIN/DF (chi-square minimum/degree of freedom) was terrible CMIN/DF = 12.459, so we looked at modification indices, which indicated that the error terms of items 2 (not being able to stop worrying) and 1 (feeling nervous or on edge), 3 (worrying too much about different things), and 4 (trouble relaxing) as well as 4 and 5 (being so restless that it is hard to sit still) were correlated, and allowing this correlation significantly improved the model fit. The correlation between the three item errors was allowed, which generated an acceptable RMSEA, CFI, and SRMS for the sample and gave improved values for CMIN/DF (Δχ2 (DF) = 128.753(3), P <.001) (**Table 4**).

**Table 4:** Confirmatory factor analysis.

	χ2 (df)	CFI	RMSEA [90% CI]	SRMR	NFI	TLI	ΔRMSEA	ΔCFI	ΔTLI	Δχ2 (df)
Single-group CFA-original factor										
Students	174.42(14)	.940	.08 [.069,.090]	.048	.936	.911	–	–	–	–
Single-group CFA (θ1, 2 free; θ3, 4 free; (θ4, 5free)										
Students	45.67(11)	.987	.042 [.030,.055]	.026	.983	.975	.03	.047	.053	128.753(3), P<.001
Multiple-group CFA models ((θ1, 2 free; θ3, 4 free; (θ4, 5free)										
Configural invariance	60.83 (22)	.985	.031 [.022,.041]	.024	.977	.971	–	–	–	–
Metric invariance	72.96 (28)	.983	.030 [.022,.038]	.035	.972	.974	.001	.002	-.003	12.13(6), P>.001
Scalar invariance	107.51 (34)	.971	.035 [.027,.042]	.030	.959	.965	.005	.012	.009	34.55(6)
τ7 free	93.84 (33)	.976	.032 [.024,.040]	.031	.964	.970	.003	.007	.004	20.88 (5) P>.001

GAD-7, Generalized Anxiety Disorder 7-item; Item 1 = “feeling nervous, anxious, or on edge”; item 2 = “not being able to stop or control worrying”; item 3 = “ Worrying too much about different things’”; item 7 = “feeling afraid as if something awful might happen”. All χ2 tests and Δχ2 were significant, P <.001.

#### Measurement invariance between the groups

The results for measurement invariance of GAD-7 are shown in previous studies, which have shown that the use of ΔCFI was associated with higher levels of metric, scalar, and residual invariance, whereas the use of Δχ2 only was associated with lower levels of scalar invariance (34); thus, in our examination of invariance, we shall include both indices. The baseline model of GAD-7 displayed a good fit (CFI = .985, RMSEA = .031, SRMR = .024, TLI = .971, NFI = .977, and with these indices, the Configural invariance was confirmed. The next step was checking metric invariance to see whether factor loadings for each GAD-7 item were equivalent across groups as confirmed total invariance (CFI = .983, RMSEA = .030, SRMS = .035, NFI = .972, TLI = .974, Δχ2 (DF) = 12.13 (6) ΔRMSEA = .001 ΔCFI = .002), and these values confirmed metric invariance suggesting equivalence. Therefore, we proceeded to test scalar invariance and we relaxed constraints for equal means in the latent variable where the male group is a reference mean and in the female group the mean for the latent variable would reflect the mean difference. The scalar level showed perfect indices except for the drop in ΔCFI = .012 (greater than 0.1), a significant rise in chi-square Δχ2 (DF) = 107.51 (34), and a decrease in TLI. Modification indices showed that the intercept of item 7 was higher in both groups, so by releasing their equality constraints, the fit model increased (CFI = .976, NFI = .964, ΔRMSEA = .003, ΔCFI = .007, 25.526 (3) P >.001 and Δχ2 (DF) 20.88 (5); these indices allowed us to confirm partial scalar invariance (**Table 4**).

#### Latent mean comparison

The comparison of latent means was based on 6 invariant items (items 1, 2, 3, 4, and 5) and the Female sample was used as a reference group. Female adolescents had a higher latent mean than male adolescents, which means that anxiety symptoms were higher in female compared with male adolescents, but the mean difference was not significant (z = .073, d = .008, P = .941).

## Discussion

Adolescence is a critical period for mental health (35), and identifying anxiety early can lead to timely interventions and support. The GAD-7 is a brief user-friendly questionnaire that assesses anxiety symptoms commonly experienced by adolescents. In our study, we examined the cross-gender measurement invariance of the GAD-7 in Rwandese adolescents from local secondary schools and reported on the prevalence of generalized anxiety symptoms severity as well as the reliability and validity of the Kinyarwanda version of GAD-7. There is a substantial difference in both genders, and the GAD-7 scores vary significantly across different groups, and female adolescent students reported higher generalized anxiety symptoms than male adolescents. These findings are consistent with previous research reports that gender differences exist in the manifestation of certain symptoms (3), and these results also correspond to previous research showing that generalized anxiety disorders are more frequent among women than among men because sex differences that occur at childhood will increase with age (36). It is important to note that these differences could be influenced by biological, psychological, and sociocultural factors (37).

In the investigation of the scale reliability, good internal consistency of this instrument suggested its ability to produce similar results under consistent circumstances; thus, the Kinyarwanda version of the GAD-7’s reliability coefficient supports its eligibility and is consistent with other studies that GAD-7 psychometric properties in adolescents (38) and other studies examining the reliability of GAD-7 in African countries (39–41) which all showed great internal consistency in different African populations. However, fewer studies conducted surveys in adolescents. The study factor loadings were >.05, this revealed a unidimensional structure. Our results indicated that the scale’s potential to evaluate generalized anxiety symptoms in adolescents was undeniably excellent, which was consistent with other studies using GAD-7 in adolescents to assess its validity properties (38, 42, 43). Furthermore, the psychometric properties of the GAD-7 in adolescents were consistent with those of studies conducted in adults (39, 44). Other studies have reported the co-occurrence of anxiety and depression frequently, and many researchers have found that the GAD-7 scale performed well not only in measuring generalized anxiety disorders but also the mixed of mixed anxiety-depression samples (40).

We used EFA as a multivariate statistical methods that identifies small numbers of hypothetical constructs such as latent variables and explain the covariation observed among a set of observable variables (41). Results in the scree plot showed that the original one-factor model of the GAD-7, which is also supported by the original validation and standardization of the GAD-7 in general populations (25), was appropriate in both groups and consistent with another study conducted in a Chinese sample of adolescents to investigate the scale’s psychometric properties in this sample (45).

In the confirmatory factor analysis for a single group, we allowed the correlation of items 2&1, 3&4, and 4&5 to improve the model fit while following the recommendations of Rolf (2014) on cutoff values of fit indices (46). The assessment of gender differences used a factorial invariance study, and fit indices indicate equivalence on the configural and metric invariance levels, which confirmed that the instrument demonstrated the same factor structure and all items have the same relationship underlying latent construct across both groups. This means that both genders interpret and respond to the items similarly emphasizing the reliability of the instrument and indicating that the relationship between the observed items and the underlying construct of anxiety is consistent across men and women. This crucial step ensures that scale measures for anxiety symptoms are consistent across genders and simultaneously enables meaningful comparisons of generalized anxiety levels between both sexes. When examining the scalar invariance, fit indices were not good in either group, and only after releasing constraints on item 7 were we able to achieve partial scalar invariance with adequate adjustment, thus achieving partial equivalent means between groups. According to the hypotheses set prior to the study, the unidimensional factor structure of GAD-7 was equivalent across both genders in Rwandese adolescents and there were no significant differences in symptoms of anxiety across groups (*P* >.001). These results implicated that there were slight differences in the item intercepts between genders and slight mean variations across genders; however, this did not compromise the overall measurement equivalence of the GAD-7. Our results consisted of a broad hypothesis that some degree of partial measurement invariance would be present for this instrument and was consistent with other meta-analyses on gender difference studies (47).

## Strengths and limitations

Among the strengths of this study, to the authors’ knowledge, this is the first study to exclusively investigate the psychometric properties of GAD-7 in a Rwandese adolescent sample size (n ≥1,000). In addition to examining the reliability and validity of this scale in Rwandese adolescents, we also provided adequate evidence on the psychometric performance of applying the Kinyarwanda version of GAD-7, which can help demonstrate the instrument’s feasibility across different sexes in young people in the Rwandese community. This data sample appropriately represented Rwandese adolescents in terms of the screening tool validation as it was highly needed and the analytic approach used contributed highly to the eligibility of the study results. This assessment tool was quick and easy to complete quickly, which makes it convenient for adolescents and healthcare providers. Its psychometric properties in this community were proven to be accurate in this age group.

Despite its vast contributions, our study is not without limitations. Although we aimed to examine the psychometric properties of the Kinyarwanda version of the GAD-7 screening tool and reported on the prevalence of generalized anxiety disorder, our sample size is not large enough to represent a whole country and due to the survey being centralized in the capital city of the country, there are emotional expression differences basing on upbringing and western culture influences on children and families in the capital city as opposed to rural areas. Thus, we only claim to understand the gender differences in symptom severity. In addition, this study was conducted at a single point, so the test–retest reliability was not conducted. Furthermore, the scale was found to be partially measurement invariant and to fulfill the prerequisite for comparison of intercepts by including only unbiased items, which undoubtedly may lead to shortcomings when it comes to interpreting cross-gender comparisons.

The GAD-7 is projected on the fact that this instrument relies on self-reporting, which entails that the accuracy of the results depends on the honesty and self-awareness of the adolescent alone. We also acknowledge that because this is merely a screening tool and not a diagnostic instrument, it cannot provide a definitive diagnosis of generalized anxiety disorder, and some anxiety disorders such as social or separation anxiety would require additional assessment tools for a comprehensive evaluation.

Therefore, we highly recommend that the confirmation of these findings be further investigated in populations living in rural areas and that trials be conducted focusing on anxiety-specific treatment as well as in clinical and primary care settings of Rwandan healthcare centers to determine the diagnostic accuracy of this screening tool.

## Conclusion

In summary, anxiety disorders are a prevalent mental health condition in Rwanda that can be attributed to various factors, including poverty, conflict, and trauma. Screening for anxiety disorders, particularly using validated tools such as GAD-7, is crucial in ensuring early identification and treatment of generalized anxiety disorder. The findings of this study confirm that the Kinyarwanda GAD-7 is a unidimensional scale with good psychometric properties among adolescents in Rwanda and an invariant construct across gender, which could be used for multiple-group comparisons. Full scalar invariance was not confirmed; this may be due to gender differences, or societal stereotypes about male behavior and hormonal differences, but further investigation across gender differences in different cultures could be recommended to further confirm these assumptions. It is also crucial to examine the universality of this scale across different age groups. There has been a representation of qualitative differences in the symptoms of anxiety among young and old adults, which were not completely assessed using the current measure of anxiety (48). This study is the first to investigate the validity and reliability of the Kinyarwanda version of GAD-7 in the Rwandan young population, which suggests that the constructs of a subject could vary in its expression across gender groups and that the measurement equivalence of this instrument should be ensured in age and cultural comparative studies.

## Data availability statement

The datasets used in the current research study are available from the corresponding authors upon reasonable request.

## Ethics statement

Ethical approval for data collection was granted by the College of Medicine and Health Sciences Institutional Review Board of the University of Rwanda (CMHS IRB). Before the questionnaires were distributed on-site, the guardians/parents of the participants signed a consent form permitting the investigator to collect data and the participants voluntarily gave verbal consent to participate in the survey. Questionnaires were filled with no further personal information apart from gender and age to ensure the anonymity of participants, and participants had the right to take part as well as revoke their data at any part of the survey. The collection of data followed regulations for the protection of human research participants.

## Author contributions

LCN: Conceptualization, Data curation, Formal analysis, Investigation, Methodology, Software, Validation, Writing – original draft, Writing – review & editing. SH: Funding acquisition, Methodology, Writing – review & editing. LK: Supervision, Writing – review & editing, Project administration.
